# Developing an Intervention to Improve Occupational Participation for Justice-Involved People with a Personality Disorder: Defining and Describing Intervention Components

**DOI:** 10.1177/0306624X211013520

**Published:** 2021-05-10

**Authors:** Catriona Connell, Vivek Furtado, Elizabeth A. McKay, Swaran P. Singh

**Affiliations:** 1 University of Warwick, Coventry, UK; 2 Birmingham and Solihull Mental Health NHS Foundation Trust, UK; 3 Edinburgh Napier University, UK; 4 Coventry and Warwickshire Partnership NHS Trust, UK

**Keywords:** personality disorder, occupational therapy, social functioning, occupational participation, participation, personality disordered offender, desistance

## Abstract

Occupational participation is undertaking personally meaningful and socially valued activities and roles. It is an important outcome for health and justice interventions, as it is integral to health and desistance. We report the third of a four-stage research project to develop an intervention to improve occupational participation for justice-involved people with a personality disorder in the community. We completed a Delphi survey to produce expert consensus on intervention components and their content, ascertain participant ratings of 28 factors for their level of influence on occupational participation, and the modifiability of the factors with this population. Thirty multi-disciplinary participants completed three survey rounds. Most factors were rated very influential, but few were considered easily modifiable. Participants agreed 121 statements describing intervention components and content. Twenty-seven statements did not reach consensus. In targeting specific factors in intervention, practitioners must balance their degree of influence with potential modifiability. The results will inform intervention manualization and modeling.

## Introduction

Occupational participation involves undertaking personally meaningful activities and roles (Taylor, 2017). When these are prosocial (valued and sanctioned by society), occupational participation is integral to health (World Health Organization, 2001), evident in the desistance process (Maruna, 2001), and associated with reduced reoffending risk (Bonta & Andrews, 2017).

In the UK, approximately 50% of people supervised by community criminal justice services and 50% to 65% of people in prison would meet diagnostic criteria for personality disorder (Brooker et al., 2012; Singleton et al., 1998). Compared to justice-involved people without a personality disorder, those with personality disorder have worse health, wellbeing, and occupational participation (Black et al., 2010; Hill et al., 2013). Justice-involved people with a personality disorder are 2.4 times more likely to reoffend compared to people without personality disorder (Yu et al., 2012).

These poor outcomes, and the association between occupational participation and both health and desistance, indicates that an intervention to improve occupational participation in the community would be advantageous. However, there is currently limited evidence for interventions (Connell et al., 2017).

This paper reports stage three of a four stage complex intervention development study, applying MRC Guidelines (Craig et al., 2008), to develop an intervention to improve occupational participation for justice-involved people with a personality disorder in the community. In describing the population for whom this intervention is designed, we use the phrase “justice-involved people” to describe people who have had interactions with the criminal justice system as a defendent and been convicted of an offence. We use the term personality disorder as understood in common medical diagnostic frameworks.

In stage one, we identified few studies of the factors that influence occupational participation for justice-involved people with a personality disorder (Connell et al., 2018), and few interventions evaluated for this purpose (Connell et al., 2017). To maximize intervention effectiveness, in stage two we determined the factors that influence occupational participation so that these could be targeted in intervention (Connell et al., 2019; Connell et al., under review).

This paper reports stage three in which we (1) determined which factors have the greatest influence on occupational participation for justice-involved people with a personality disorder, (2) determined the degree to which these factors are modifiable, and (3) defined and described the components of an intervention.

The results will inform stage four of the project, when we will manualize the intervention and model potential mechanisms of action.

## Method

We employed a three round online Delphi survey with a panel of multidisciplinary experts. We selected a consensus development method due to the lack of research on effective interventions and the diverse range of practitioners supporting this population. A Patient and Public Involvement (PPI) group informed the study design.

### Participants

We defined experts as people with research or practice experience working with justice-involved people with a personality disorder in the community. Inclusion criteria for practitioners were: qualified health, social care or criminal justice professional, or a criminal justice practitioner with at least 12 months experience; and had a minimum 12 months experience working with justice-involved people with a personality disorder in the community. The inclusion criterion for researchers was that they had published on health, social or desistance outcomes for justice-involved people with a personality disorder in the community.

### Sampling and Recruitment

We adopted purposive sampling followed by snowball sampling to access the small, dispersed, and hard to locate population (Given, 2008). We aimed to recruit 30 participants. This aimed to generate a sample with enough participants from the same professional background to make the consensus process meaningful, whilst being mindful of the potential low number of expert participants.

We identified potential participants through authorship of publications reviewed during systematic reviews, presentation at conferences, membership of national special interest groups, the Offender Personality Disorder (OPD) Pathway teams in England, and the national register of OPD Services. These participants nominated others who met inclusion criteria.

We approached 34 potential participants by email. One declined further involvement and three did not respond. The sample thus consisted in 30 participants. We supplied full information in a study information sheet about participant rights, including to withdraw and how to contact the researcher, which was also presented on commencing the online survey.

#### Data collection and analysis

We piloted survey questions with members of the PPI group, collecting data via an online survey platform (Qualtrics, 2018). The structure differed between rounds. We concluded the Delphi process after three rounds to sustain a good response rate from participants from different professional backgrounds, maintain comparability with other similar surveys and reduce participant burden (Hasson et al., 2000).

We sent an individual link to round one via email to all participants. Those who completed a round were invited to participate in the following round by email with another individual link. Each survey was available for 4 weeks. Two standardized email reminders and additional contact with participants by email and telephone maximized participation rates and reduced attrition bias.

We calculated response rate after each round and overall. The pilot survey and methods for rounds one to three are described below. We conducted analysis using SPSS (IBM Corp, Released 2016), MAXQDA (VERBI Software, 2017), and Microsoft Excel.

#### Pilot survey

PPI group members piloted the survey questions for round one and an example of what may be included in round two, as round two was informed by round one responses. Data collected were appropriate to the study aims and sufficiently detailed. The survey software functioned well. Participant feedback informed a reduction in the length and complexity of round one questions to increase practitioner completion rates. One person piloted the revised version and confirmed it was feasible for practitioners to complete.

#### Round one data collection

We presented 26 factors initially identified as influencing occupational participation (Connell et al., under review) to participants along with its descriptor. Participants rated how much each factor influenced occupational participation on a 0 to 10 Likert scale, where 0 was “no influence” and 10 was “critical influence.” Participants then rated the same factors for their potential for modification through intervention on a 0 to 10 Likert scale, where 0 was “impossible to change” and 10 was “simple to change.” Participants were invited to make free-text comments on this and in all other elements of each round.

Participants described interventions they delivered, or thought would be best practice to deliver, to address occupational participation and its influencing factors. Questions prompted participants for the details required to standardize the intervention for practice and research (e.g., duration, practitioner skills) (Carroll & Nuro, 2002; Hoffmann et al., 2014; Möhler et al., 2015).

#### Round one data analysis

We analyzed the ratings of importance and modifiability using descriptive statistics and tabulated these in order of most to least influential, and most to least modifiable by mean score. We converted qualitative descriptions of interventions into statements. Where possible, intervention content and/or components were paired with the factor/s it aimed to address. For example, “a time-use tool is an effective way of increasing knowledge about the function of time use.” We grouped similar statements where they described a similar intervention function (e.g., assessment) and then combined those that referred to the same or similar intervention content. We analyzed free text responses by coding these and grouping them where appropriate.

#### Round two data collection

We presented the factors in order of influence and modifiability based on mean scores from round one and invited participants to comment. Participants then rated their agreement with each statement developed from round one on a five-point Likert scale. Options were “strongly disagree,” “somewhat disagree,” “neither agree nor disagree,” “somewhat agree,” and “strongly agree.” Free text comments served as a quality check on the round one analysis, as participants could highlight if they felt their perspective was not represented.

#### Rounds two data analysis

We accepted a statement where 75% of participants agreed or strongly agreed, consistent with Delphi studies on intervention development in mental health and personality disorder treatment (Cook & Birrell, 2007; Kelly et al., 2010; Tetley et al., 2012). We returned statements that did not reach this consensus level to participants in round three with a graph summarising of all participants ratings. We made adaptations to statement wording, combined statements and included new statements in round three based on analysis if the free text responses.

#### Round three data collection

For each statement which did not reach consensus in round two, we showed participants a bar chart informing them of responses given by all participants. Participants then re-rated the statements on the same five-point Likert scale.

#### Round three data analysis

We accepted a statement where 75% agreement was achieved. We hypothesized that there may be higher agreement levels if results were analyzed by participants’ professional backgrounds. Thus, we compared consensus agreement between health and criminal justice professionals on statements that did not reach consensus overall. However, statements were not accepted if only one professional group reached consensus.

## Results

### Participants

Participants were from criminal justice (probation officer/offender manager/police officer/social work) or health (occupational therapy/psychology) backgrounds. Table 1 shows the participant demographics collected at round one.

**Table 1.** Participant Demographics.

	*N* (%)
Professional background	
Health care	13 (46)
Criminal justice	13 (46)
Clinical academic	2 (8)
Length of experience with PDOs in community	
Under 1 year	2 (7)
1–5 years	12 (43)
5+ years	13 (46)
Academic only	1 (4)
Gender	
Male	8 (29)
Female	19 (68)
Other	1 (3)
Age	
Under 25	0 (0)
25–40	9 (32)
40+	18 (64)
Decline to answer	1 (3)

### Response Rate

Twenty-eight participants (93%) responded to round one, 24 (80%) in round two and 21 (70%) in round three. Table 2 shows the response rate for each round. One respondent’s data was not properly recorded in round one. We extended the invitation to round two due to their engagement with the research.

**Table 2.** Response Rate by Round.

Round	*N* (%) completion in round	*N* (%) completion all rounds	*N* (%) drop out in round	*N* (%) drop out overall
Round one	28/30 (93)	28/30 (93)	2 (7)	2 (7)
Round two	24/28 (86)	24/30 (80)	4 (14)	6 (20)
Round three	21/24 (88)	21/30 (70)	3 (13)	9 (30)

### Factors Influence on Participation

In round one, participants rated 26 factors. In several free text responses, participants proposed additional factors. We appraised these and compared with previous mixed methods work (Connell et al., under review) and accordingly added two factors: “environmental resources” (including community amenities) and “financial stability.” These were rated in round two resulting in a total of 28 factors rated for their level of influence on occupational participation and their modifiability.

All the factors were scored above five on average for influence, whereas only eight factors were scored above five on average for modifiability. Six of the eight more modifiable factors were in top half for their influence on occupational participation: “safe home,” “self-efficacy in activity,” “sustains routine,” “problem-solving,” “role,” and “adaptability.”

Table 3 shows the factors in descending order by mean score for degree of influence on occupational participation. The factor with the highest mean is considered the most influential factor by expert consensus. Column four indicates the mean score for modifiability and column five shows its ranking compared to other item scores for modifiability. The factors in the top half for influence and scoring above 5/10 for modifiability are indicated.

**Table 3.** Factor Ratings.

Factor	Importance (mean)	Importance (rank)	Modifiability (mean)	Modifiability (rank)
Emotional stability	8.07	1	4.53	18
Relating to people	7.85	2	4.85	13
Safe home^a^	7.81	3	5.11	7
Environmental resources	7.48	4	4.00	23
Perceptions of social judgment	7.37	5	4.15	21
Self-efficacy in activity^a^	7.37	5	5.37	3
Past intrudes	7.33	7	3.89	24
Sustains routine^a^	7.30	8	5.81	1
Self-efficacy in social settings	7.22	9	4.96	10
Problem-solving^a^	7.22	9	5.26	5
Role^a^	7.15	11	5.38	2
Past belonging	7.15	12	2.93	28
Adaptability^a^	7.07	13	5.19	6
Past mastery	6.96	14	4.30	20
Prosocial interests	6.96	15	5.11	7
Social validation	6.93	16	4.52	19
Knowledge	6.89	17	4.85	13
Responsibility	6.89	17	4.93	11
Goals	6.85	19	5.37	3
Financial stability	6.71	20	4.14	22
Manages attentional demands	6.67	21	4.74	15
Past routine	6.59	22	3.23	26
Conversational skills	6.56	23	4.93	11
Non-verbal skills	6.33	24	4.70	17
Past role	6.33	24	3.19	27
Criminal record and disclosure	5.89	26	3.63	25
Physical capacity	5.70	27	5.00	9
Emotional/sensory feedback	5.15	28	4.74	15

a Indicates a factor in the top half for influence and scoring above 5/10 for modifiability.

Participants made the ratings in general terms rather than in reference to a specific individual. For an individual, the relative importance and modifiability of the factors would vary.

### Participant Comments on Factors

Two participants commented to indicate agreement with the importance of the new factors (environmental resources, financial stabilty), but also how challenging they could be to modify. One participant was uncertain about physical capacity, and their ability to assess its impact on occupational participation. This participant also wrote in detail about the wider environmental challenges of accommodation and criminal record disclosure for this group whose convictions were often never “spent.”

### Statements Reaching Consensus

We analyzed practitioners’ descriptions from round one about the best way to intervene to improve occupational participation, to produce 150 statements. We initially clustered these into groups according to the intention of the practitioner in the description (e.g., all statements describing assessment were grouped together). Consensus was achieved on 110 statements, which were removed from the next round. We reviewed, amended, and condensed the remaining 40 statements to 38 before presenting these back to participants. A further 11 statements reached consensus in round three.

We grouped the statements according to similarity in either describing a specific action or overall principles as follows: intervention principles, therapeutic relationship, practitioner behaviors, assessment, formulation, education and goal setting, increasing participation, and endings. In the next stage of our intervention development project, these initial groupings will be further analyzed and used to specify and describe distinct intervention components. Components will have a defined function, clear description and a explanation for how the component effects change using the Model of Human Occupation.

### Statements Not Reaching Consensus

We analyzed the 27 statements that failed to achieve consensus after round three. These referred to time limiting aspects of the intervention, telephone contact with participants, introducing activity in the early stages of intervention, self-assessments, practitioner disclosure, and integrating digital technology within the intervention. Most comments in round three related to the need for personalization and barriers to internet-enabled technology. Some felt technology use was impossible due to probation practice requirements to manage risk, and a lack of digital infrastructure on the premises. Some were concerned the person may not be able to afford the technology. Others highlighted legal restrictions on internet use for people convicted of certain sexual offenses.

Among these 27 statements, two achieved consensus when considering only the responses of participants from a criminal justice background, both of which referred to time limiting aspects of intervention. Four statements achieved consensus when considering only the participants from a health background which related to individual tailoring, ways of concluding sessions and reviewing progress against agreed goals at six weekly intervals.

To determine if there was consensus *disagreement* by professional background, we identified statements where 50% or more participants strongly disagreed or somewhat disagreed. There was disagreement by 50% of participants from a criminal justice background, and 54% of participants from a health care background on the statement, “Limiting disclosure about practitioner experiences is an effective way to build a therapeutic relationship.”

## Discussion

In a three round Delphi survey, participants from a range of disciplinary backgrounds reached consensus agreement on 121 statements describing the content of an intervention to improve occupational participation. Twenty-seven statements were rejected. The agreed statements will inform a further study to develop a manualized intervention with distinct and well described components to improve occupational participation for justice-involved people with a personality disorder in the community.

Delphi surveys have no defined optimum response rate. By retaining 70% of participants, our response rate is comparable to e-Delphi surveys with geographically spread professionals that have gone on to inform intervention development (Kelly et al., 2008, 2009, 2010; Langlands et al., 2008; Santos et al., 2018). Our results will therefore be of value in the next stage of intervention development.

Several factors thought most influential were rated as difficult to modify (e.g., emotional stability was first for influence, but 18th for modifiability). Low ratings on modifiability across factors may reflect the professional backgrounds of the participants. Less than half were occupational therapists, who have specific expertise in addressing occupational participation. A sample of all occupational therapists may have yielded different results. Alternatively, criminal justice practitioners (46%) may have a more realistic appraisal of a factor’s modifiability given their experience working with those complelled to attend interventions in community justice settings. However, it is also possible that low modifiability scores reflect the complexity of modifying occupational participation in this context.

Two of the five most influential factors refer to the environment. Yet, free-text responses and modifiability ratings indicated that environmental factors are not considered easy to change. “Safe home” was the most modifiable but still only scored 5.11/10 on average for modifiability. Nonetheless given its importance, supporting someone to access a home in which they feel safe is likely a critical factor to address before asking for engagement in an intervention that encourages further changes.

Practitioners must balance the importance of a factor with its potential for modification to target efforts towards those most likely to achieve improvements in occupational participation. “Safe home,” “self-efficacy in activity,” “sustains routine,” “problem-solving,” “role,” and “adaptability” were rated between five and six for modifiability and were in the top 14 most influential factors. These may be useful intervention targets.

Cook and Birrell (2007) used a Delphi study of occupational therapists to specify the content of an occupational therapy intervention for people with psychosis in the community. Although they do not define the intended outcome of the intervention, occupational participation is central focus of occupational therapists in mental health services. Areas of Cook’s (2006) intervention specification overlap with the preliminary groupings in this study, including assessment, goal setting, and considering endings. Details within the “action” component reflect several statements agreed by participants in this study. These similarities likely reflect the familiarity of participants in both studies with occupational therapy process descriptions (e.g., Creek, 2003).

In contrast, “analysis” was only a small part Cook’s (2006) specification, compared to the strong endorsement of statements referring to formulation by participants in this study. This likely reflects differences in professional backgrounds, as the practice of formulation is far more established in psychology than occupational therapy, hence its emphasis in this study. Therapeutic relationships received far greater emphasis by participants in this study. Relative challenges in developing and sustaining therapeutic relationships have been described when working with people whose experience of “helping” others has not always been positive, or who are suspicious of the intentions of criminal justice staff (Craissati et al., 2020). As a result, participants may more consciously attend to this in their practice.

The importance of a therapeutic relationship is well documented as a common factor in the effectiveness of psychotherapy (Wampold, 2015) and mental health occupational therapy (Wimpenny et al., 2014), and is argued to be an essential consideration in probation services (Burnett & McNeill, 2005). Participants in this study took responsibility for working on a therapeutic relationship *in order to support occupational participation*. Taylor (2008) argued that the therapeutic relationship requires different consideration in interventions to modify occupational participation, where it is used intentionally as a tool to improve occupational participation, rather than the relationship itself being the focus.

Tetley et al. (2012) used a Delphi study to identify essential elements to facilitate therapy engagement with people with personality disorder. As in this study, participants identified external factors, such as environmental stressors like housing, or having a supportive peer group. The interaction between influencing factors supports the characterization of an intervention to improve occupational participation as complex.

A preference for flexibility resulted in statements referring to time limiting aspects of the intervention failing to achieve consensus. Individualized and person-centered intervention is a core ethical principle of occupational therapy (College of Occupational Therapists, 2015) and psychology (British Psychological Society, 2018). However, intervention standardization is required for good quality effectiveness research, which includes specification of timings.(Hoffmann et al., 2014; Möhler et al., 2015). It may be more appropriate to determine if a component is delivered successfully based on observable change or documented task completion, with timing estimates made as the intervention is evaluated and refined.

Most comments in round three related to digital technology. Using the internet is an essential aspect of modern life (Larsson-Lund, 2018). It is integral to occupational participation in the UK, enabling communication and connection, employment searches, price comparisons to support budgeting, and access to information about leisure (UK Government, 2018). Inability to use internet-enabled technologies may compound the health, economic and social divide experienced by socially disadvantaged groups (Farooq et al., 2015). The pace of technology development coupled with the “digital divide” between prison and the community results in people being released from prison ill-equipped for the demands of modern society, exposed for their lack of knowledge and more vulnerable to social exclusion, even where a sentence is relatively short (Enyon & Geniets, 2012; Reisdorf & Jewkes, 2016; Reisdorf & Rikard, 2018). Whilst restricting internet access provides an option for reducing some risks, it exposes people to others. Practitioners must recognize that lack of digital skills and access may be a barrier to occupational participation and consider this within any intervention. More discussion and debate around the issue of digital exclusion in community justice settings is needed.

The results of this study will inform manualization and modeling of a multi-component complex intervention to improve occupational participation for justice-involved people with a personality disorder in the community. Future research should determine whether the intervention is feasible to deliver in practice and the optimal design for an effectiveness study.

### Strengths and Limitations

Delphi surveys are ideal for developing consensus in areas where research is limited, but they carry a risk of bias and groupthink depending expert selection. All participants were in England, and may have been influenced by current service provision and its values. We used purposive sampling to recruit experts from a range of disciplinary backgrounds to capture perspectives based on different theoretical and evidence bases. Disagreement remained after round three, indicating our varied sample mitigated some of the bias risk. Nonetheless, an international sample may have come to different conclusions.

Including justice-involved people with a personality disorder as participants would have been a powerful additional perspective. We discussed this with the PPI group. We concluded this would be better addressed with different survey questions to optimize its value for participants and researchers, but we could not achieve that within this study. This is an important gap requiring attention.

We acknowledge that in practice, few people will have a formal personality disorder diagnosis. Having based this and previous studies in the practice reality (Connell et al., 2019), we anticipate utility of the intervention with people screened as likely to have personality disorder.

## Conclusion

For justice-involved people with a personality disorder living in the community, multidisciplinary experts rated 28 factors for their level of influence on occupational participation. No factors were considered easy to modify, all scoring below six out of ten on average. Practitioners must evaluate the relative gains of targeting factors of greater or lesser influence, and their degree if modifiability, but should remain aware of individual needs.

When considering how best to intervene to improve occupational participation for justice-involved people with a personality disorder in the community, the expert panel reached consensus on 121 statements describing the content of intervention components. Agreement was divided on digital technology, which raises important considerations about the balance between risk management and optimizing occupational participation. These results provide a basis to specify the components of an intervention to improve occupational participation.
